# FINANCIAL LITERACY, RETIREMENT, AND BECOMING FINANCIALLY CAPABLE IN A DEVELOPING COUNTRY

**DOI:** 10.1093/geroni/igz038.2149

**Published:** 2019-11-08

**Authors:** Julian G McKoy Davis, Denise Eldemire-Shearer

**Affiliations:** Mona Ageing & Wellness Centre, Kingston, Jamaica

## Abstract

Worldwide, 3.5 billion (67%) of older adults do not understand basic financial concepts. In the Caribbean, families and professionals alike are struggling to assist and financially support a rapidly ageing population. The concept of children as “pension guarantee” is widely practised throughout the Caribbean, but factors such as modernization, urbanization, migration, and a shift in societal norms pertaining to familial responsibilities, have left many older persons financially fending for themselves. Less than half of all Jamaicans have adequate retirement funds. People with limited financial resources can and do save, but often use strategies that are not advantageous. The stark reality of low pension coverage among older Jamaicans and austere working conditions among public and some private sector workers indicate growing economic, financial, and societal challenges. Jamaica’s rapidly growing ageing population has resulted in both a workforce and a retirement financial crisis. This symposium provides an overview of the current policy and economic issues. We describe the design, implementation, and evaluation of an economic program to increase financial literacy and planning of adults nearing retirement age. To increase financial retirement literacy of Jamaicans with limited educational and financial resources, a peer-to-peer program was developed using a culturally adapted version of the FDIC Money Smart Program. A peer-to-peer approach has the potential to strengthen financial literacy and enhance retirement planning behaviours of underserved adults and avoid further burdening an already limited workforce. This project’s findings can inform policy, shape program development, and guide implementation of similar programs in low resource countries.

